# Comparison of Risk of Recrudescent Fever in Children With Kawasaki Disease Treated With Intravenous Immunoglobulin and Low-Dose vs High-Dose Aspirin

**DOI:** 10.1001/jamanetworkopen.2019.18565

**Published:** 2020-01-03

**Authors:** Brooks Platt, Emily Belarski, John Manaloor, Susan Ofner, Aaron E. Carroll, Chandy C. John, James B. Wood

**Affiliations:** 1 Indiana University School of Medicine, Indianapolis; 2 Indiana University Health, Indianapolis; 3 Ryan White Center for Pediatric Infectious Diseases and Global Health, Indiana University School of Medicine, Indianapolis; 4 Department of Biostatistics, Indiana University School of Medicine, Indianapolis; 5 Center for Pediatric and Adolescent Comparative Effectiveness Research, Indiana University School of Medicine, Indianapolis

## Abstract

**Importance:**

Timely initiation of intravenous immunoglobulin plus aspirin is necessary for decreasing the risk of recrudescent fever and coronary artery abnormalities in children with Kawasaki disease (KD). The optimal dose of aspirin, however, remains unclear.

**Objective:**

To evaluate whether initial treatment with low-dose compared with high-dose aspirin in children with KD is associated with an increase in fever recrudescence.

**Design, Setting, and Participants:**

A retrospective cohort study of 260 children with KD at Riley Hospital for Children, Indianapolis, Indiana, between January 1, 2007, and December 31, 2018, was conducted. Children aged 0 to 18 years with a first episode of KD, identified by *International Classification of Diseases, Ninth Revision* and *International Statistical Classification of Diseases and Related Health Problems, Tenth Revision* diagnosis codes treated within 10 days of symptom onset with high-dose intravenous immunoglobulin plus aspirin were eligible. Patients who received an alternative diagnosis, experienced a second episode of KD, did not receive intravenous immunoglobulin plus aspirin for initial treatment, were not treated within 10 days of symptoms, or had incomplete records were excluded.

**Exposures:**

High-dose (≥10 mg/kg/d) or low-dose (<10 mg/kg/d) aspirin therapy.

**Main Outcomes and Measures:**

The primary outcome was recrudescent fever necessitating retreatment of KD. The secondary outcomes were coronary artery abnormalities and hospital length of stay.

**Results:**

Among the 260 patients included, the median (interquartile range) age was 2.5 (1.6-4.3) years, 103 (39.6%) were girls, 166 (63.8%) were non-Hispanic white, 57 (21.9%) were African American, 22 (8.5%) were Asian, 11 (4.2%) were Hispanic, and 4 (1.5%) were of unknown race/ethnicity. One hundred-forty-two patients (54.6%) were treated with low-dose aspirin. There was no association between recrudescent fever and aspirin dose, with 39 children (27.5%) having recrudescent fever in the low-dose group compared with 26 children (22.0%) in the high-dose group (odds ratio [OR], 1.34; 95% CI, 0.76-2.37; *P* = .31), with similar results after adjusting for potential confounding variables (OR, 1.63; 95% CI, 0.89-2.97; *P* = .11). In a subset analysis of 167 children with complete KD, however, there was nearly a 2-fold difference in the odds of recrudescent fever with low-dose aspirin (OR, 1.87; 95% CI, 0.82-4.23; *P* = .14), although this difference did not reach statistical significance. In addition, no association was identified between treatment group and coronary artery abnormalities (low-dose, 7.4% vs high-dose, 9.4%; OR, 0.86; 95% CI, 0.48-1.55; *P* = .62) or median (interquartile range) length of stay (3 [3-5] days for both groups; *P* = .27).

**Conclusions and Relevance:**

In this study, low-dose aspirin for the initial treatment of children with KD was not associated with fever recrudescence or coronary artery abnormalities. Given the potential benefits, further study of low-dose aspirin to detect potentially clinically relevant outcome differences is warranted to inform treatment decisions and guideline development.

## Key Points

**Question:**

Is initial treatment with low-dose aspirin, along with intravenous immunoglobulin, for children with Kawasaki disease associated with an increase in recrudescent fever?

**Findings:**

In this cohort study of 260 children with Kawasaki disease, initial treatment with low-dose aspirin was not associated with higher odds of fever recrudescence compared with children treated with high-dose aspirin. In addition, no association was identified between low-dose aspirin and coronary artery abnormality or length of stay.

**Meaning:**

Given the potential benefits and similar outcomes, further investigation into the use of low-dose aspirin in conjunction with intravenous immunoglobulin for the initial treatment of children with Kawasaki disease is warranted.

## Teaser

This cohort study examines the incidence of recrudescent fever during use of low-dose vs high-dose aspirin in conjunction with intravenous immunoglobin in children with Kawasaki disease.

## Introduction

Kawasaki disease (KD) is an acute systemic vasculitis of unknown cause with the potential for serious sequelae, including coronary artery abnormalities, in 15% to 25% of untreated patients.^1,2^ With timely initiation of treatment, however, the risk of coronary artery abnormalities is greatly reduced. In the United States, the standard of care for initial treatment of KD is high-dose intravenous immunoglobulin, along with acetylsalicylic acid (aspirin).^1^ Although the role and dose of intravenous immunoglobulin, 2 g/kg, are well established and have been shown to significantly reduce the risk of coronary artery abnormalities,^3,4,5,6,7,8^ the optimal dose of aspirin is unclear, resulting in significant practice variation.^1,5,9,10^

In higher doses, aspirin is used as an anti-inflammatory agent, while at lower doses it has antithrombotic effects. Both of these actions are thought to be useful in the initial stages of KD. Given concerns for adverse drug effects with aspirin,^11^ especially at higher doses, questions regarding the utility and optimal dose of aspirin have arisen. Although multiple studies have shown no difference in coronary artery abnormality risk between low- and high-dose aspirin,^9,12,13^ the effect on recrudescent fever, especially in the US population, is understudied.^3,4,14^ We therefore sought to evaluate whether the dose of aspirin was associated with recrudescent fever, hypothesizing that, given its the anti-inflammatory properties, high-dose aspirin would be associated with a decrease in recrudescent fever compared with low-dose aspirin in children with KD.

## Methods

### Study Design and Population

This was a single-center, retrospective cohort study of children aged 0 to 18 years with KD who were treated at Riley Hospital for Children, Indianapolis, Indiana, between January 1, 2007, and December 31, 2018. Potential study participants were identified by *International Classification of Diseases, Ninth Revision* (*ICD-9*) and *International Statistical Classification of Diseases and Related Health Problems, Tenth Revision* (*ICD-10*) (446.1 and M30.3, respectively) diagnosis codes and were included in the study if they were diagnosed with a first episode of KD and treated within 10 days of symptom onset with high-dose (2 g/kg) intravenous immunoglobulin plus aspirin. Patients were excluded if they had an alternative diagnosis, experienced a second episode of KD (recurrent KD), did not receive high-dose intravenous immunoglobulin plus aspirin or received adjunctive medicine (eg, corticosteroids) for the initial treatment of KD, were not treated within 10 days of symptom onset, or had incomplete records. All records were reviewed for eligibility by the study team. The study was approved by the Indiana University Institutional Review Board, with a waiver of informed patient consent as a retrospective review and because of the feasibility of obtaining informed consent from the participants. This study followed the Strengthening the Reporting of Observational Studies in Epidemiology (STROBE) reporting guideline.

### Treatment Groups and End Points

Patients who met inclusion criteria were placed into 2 groups: high-dose (≥10 mg/kg/d, which encompasses both high- and medium-dose^1^) or low-dose (<10 mg/kg/d) aspirin based on the initial dose of aspirin given for the treatment of KD. Patients treated with medium-dose aspirin were grouped with high-dose because both doses are used to treat inflammation.

The primary end point was the recrudescence of fever, defined as a temperature of 38 °C or higher orally or rectally or 37.5 °C axillary necessitating retreatment for KD within 14 days of initial treatment. The secondary end points of this study were coronary artery abnormality seen at any time during follow-up as well as hospital length of stay.

### Data Collection

Using the electronic medical record, patients were screened for eligibility; if inclusion criteria were met, the following data were collected: demographic characteristics (age, race/ethnicity [defined by the investigators after medical record review], and sex), symptoms before admission (fever, exanthem, conjunctivitis, mucous membrane changes, adenopathy, extremity swelling, and peeling), fever duration before treatment, dose of intravenous immunoglobulin, brand of intravenous immunoglobulin, dose of aspirin, recrudescence of fever necessitating retreatment of KD, type of retreatment, laboratory results (white blood cell count, hemoglobin level, platelet count, and erythrocyte sedimentation rate and C-reactive protein, aspartate aminotransferase, alanine aminotransferase, albumin, and bilirubin levels), echocardiogram results (initial and follow-up), and dates of hospital admission and discharge.

### Statistical Analysis

Data were summarized for the high- and low-dose aspirin groups. Continuous variables were tested for a difference between groups by means of the independent *t* test for normally distributed data and Mann-Whitney test or Wilcoxon rank sum test for nonnormally distributed data. Categorical variables were tested by means of χ^2^ or Fisher exact test. Bivariate logistic models were fit for the primary outcome of recrudescent fever. A multivariable regression model was built by including the indicator variable for low-dose aspirin and all variables that were significant at the .30 level in bivariate models. Except for the term *low-dose aspirin*, which we required to remain in the model, backward variable selection was used to remove nonsignificant variables until all remaining variables were significant at the .05 level with 2-tailed, unpaired testing.

Similar analytic methods were used for the analysis of the secondary outcome of abnormal echocardiogram. Plots examining the linearity assumption of the continuous independent variables with the logit of abnormal echocardiogram showed nonlinear patterns. To address this outcome, continuous variables were coded as categorical so that the resulting associations were linear with the logit of the dependent variable.

Length of stay was log transformed because the data were right skewed. Bivariate regression models were fit to the log of length of stay. Residual plots were examined to assess model assumptions of homogeneity of variance and normality (eFigure in the Supplement). When building a multivariable model, a similar approach for variable selection was used for length of stay. Data analysis was performed with SAS, version 9.4 (SAS Institute Inc).

## Results

### Population Characteristics

A total of 673 patients were identified by *ICD-9* and *ICD-10* codes and screened for eligibility, and 413 of those patients were excluded. Of the excluded patients, 135 received an alternative final diagnosis, 184 had absent or incomplete medical records, 49 had no record of KD, 42 received treatment after 10 days of fever or received treatment other than intravenous immunoglobulin, 2 g/kg, plus aspirin, and 3 were excluded owing to having recurrent KD.

Table 1 presents the characteristics of the study population. Among the 260 patients included, the median (interquartile range [IQR]) age was 2.5 (1.6-4.3) years, 103 (39.6%) were girls, 166 (63.8%) were non-Hispanic white, 57 (21.9%) were African American, 22 (8.5%) were Asian, 11 (4.2%) were Hispanic, and 4 (1.5%) were of unknown race/ethnicity. The median (IQR) dose of intravenous immunoglobulin was 1.99 (1.92-2.07) mg/kg. Of the 260 included patients, 142 patients (54.6%) received low-dose aspirin (<10 mg/kg/d) and 118 patients (45.4%) received high-dose aspirin (≥10 mg/kg/d). The median (IQR) daily dose of aspirin in the low-dose group was 4.4 (3.6-5.1) mg/kg; the median (IQR) daily dose in the high-dose group was 75.5 (41.7-86.1) mg/kg. Most children in the low-dose aspirin group received 3 to 5 mg/kg/d and were treated earlier than 2016, while most in the high-dose group received greater than 50 to 100 mg/kg/d and were treated after 2015 (Table 2 and Figure). Treatment groups were similar with respect to age, sex, race/ethnicity, fever duration prior to intravenous immunoglobulin treatment, coronary artery abnormality at diagnosis, white blood cell count, hemoglobin level, platelet count, erythrocyte sedimentation rate, and alanine and aspartate aminotransferase levels. Most patients in both groups received Privigen brand intravenous immunoglobulin. In addition, clinical presentation was similar between the 2 groups with respect to the proportion of exanthem, mucous membrane changes, cervical lymphadenopathy, extremity changes, nonpurulent conjunctivitis, extremity peeling, and groin peeling (Table 3). The high-dose group, however, exhibited a higher proportion of patients with incomplete KD compared with the low-dose group at 44.1% (n = 52) and 28.9% (n = 41), respectively (*P* = .01), and had a higher median albumin level (3.6 vs 3.1 g/dL [to convert to grams per liter, multiply by 10], *P* < .001). There were no adverse effects due to aspirin in either treatment group.

**Table 1.** Characteristics of Study Population

Characteristic	Overall (N = 260)	Low-Dose (n = 142 [54.6%])	High-Dose (n = 118 [45.4%])	*P* Value^a^
Demographic				
Age, y				
Median (IQR)	2.5 (1.6-4.3)	2.4 (1.5-4.4)	2.7 (1.7-4.2)	.66
<1 y, No. (%)	40 (15.4)	24 (16.9)	16 (13.6)	.46
Sex, No. (%)				
Female	103 (39.6)	61 (43.0)	42 (35.6)	.23
Male	157 (60.4)	81 (57.0)	76 (64.4)	
Race/ethnicity, No. (%)				
Non-Hispanic white	166 (63.8)	98 (69.0)	79 (66.9)	.94
African American	57 (21.9)	30 (21.1)	27 (22.9)	
Asian	22 (8.5)	12 (8.5)	10 (8.5)	
Unknown race	4 (1.5)	2 (1.4)	2 (1.7)	
Hispanic	11 (4.2)	9 (6.3)	2 (1.7)	.56
Unknown ethnicity	4 (2.7)	2 (1.8)	2 (5.1)	
Clinical				
Days of fever, median (IQR)^b^	6.0 (5.0-8.0)	6.0 (5.0-8.0)	6.0 (5.0-8.0)	.94
Intravenous immunoglobulin brand, No. (%)				
Privigen	159 (61.1)	91 (64.1)	68 (57.6)	
Gammagard	34 (13.1)	19 (13.4)	15 (12.7)	
Octagam	8 (3.1)	8 (5.6)	0	
Carimune	3 (1.2)	3 (2.1)	0	
Other or unknown	59 (22.7)	21 (14.8)	38 (32.2)	
Aspirin dose, median (IQR), mg/kg/d	6.5 (4.4-56.5)	4.4 (3.6-5.1)	75.5 (41.7-86.1)	<.001
Incomplete KD	93 (35.8)	41 (28.9)	52 (44.1)	.01
Coronary artery abnormality at diagnosis	58 (22.3)	30 (21.1)	28 (23.7)	.62
Laboratory values, median (IQR)				
WBC, /μL	14.5 (10.8-18.2)	13.8 (1.6-18.4)	15.0 (11.0-17.8)	.34
Hemoglobin, g/dL	11.0 (10.3-11.6)	11.0 (1.3-11.5)	11.0 (10.3-11.7)	.56
Platelets, ×10^3^/μL	355.0 (292.0-450.0)	352.0 (282.0-439.0)	357.0 (297.0-468.0)	.36
ESR, mm/h	65.0 (47.0-87.0)	63.0 (52.0-82.0)	65.0 (42.0-93.0)	.72
CRP, mg/L	12.5 (6.2-20.1)	1.0 (4.8-18.4)	14.7 (7.4-21.4)	.005
Albumin, g/dL	3.3 (2.9-3.7)	3.1 (2.7-3.6)	3.6 (3.1-3.8)	<.001
AST, U/L	36.0 (25.0-60.5)	35.0 (26.0-59.0)	38.0 (23.0-61.5)	.98
ALT, U/L	44.0 (17.0-99.0)	45.0 (19.0-88.0)	42.0 (17.0-110.0)	.47

Abbreviations: ALT, alanine aminotransferase; AST, aspartate aminotransferase; CRP, C-reactive protein; ESR, erythrocyte sedimentation rate; IQR, interquartile range; KD, Kawasaki disease; WBC, white blood cell count.

SI conversion factors: To convert albumin to grams per liter, multiply by 10; ALT to microkatals per liter, multiply by 0.0167; AST to microkatals per liter, multiply by 0.0167; CRP to nanomoles per liter, multiply by 9.524; hemoglobin to grams per liter, multiply by 10; platelets to ×10^9^ per liter, multiply by 1; and WBC to ×10^9^ per liter, multiply by 0.001.

^a^ *P* value determined using independent *t* test for normally distributed data and Mann-Whitney test for nonnormally distributed data.

^b^ Days of fever before treatment with intravenous immunoglobulin.

**Table 2.** Aspirin Therapy Stratified by Dose

Aspirin Group	Patients, No. (%)
Low dose, mg/kg/d	
No.	142
<3	13 (9.2)
3-5	89 (62.7)
>5-10	40 (28.2)
High dose, mg/kg/d	
No.	118
>10-30	6 (5.1)
>30-50	42 (35.6)
>50-100	65 (55.1)
>100	5 (4.2)

**Figure.**
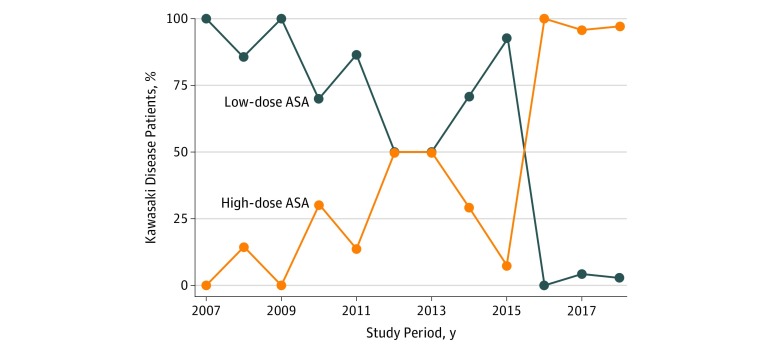
Aspirin Dosing By Year Median dose of initial aspirin therapy during the study period. ASA indicates acetylsalicylic acid.

**Table 3.** Clinical Symptoms by Group

Clinical Presentation	Patients, No. (%)			*P* Value
	Overall (N = 260)	Low Dose (n = 142)	High Dose (n = 118)	
Exanthem	239 (91.9)	133 (93.7)	106 (89.8)	.26
Mucous membrane changes	214 (82.3)	120 (84.5)	94 (79.7)	.31
Swelling	164 (63.1)	96 (67.6)	68 (57.6)	.10
Cervical lymphadenopathy	103 (39.6)	54 (38.0)	49 (41.5)	.57
Conjunctivitis	223 (85.8)	124 (87.3)	99 (83.9)	.43
Extremity peeling	25 (9.6)	15 (10.6)	10 (8.5)	.57
Groin peeling	41 (15.8)	20 (14.1)	21 (17.8)	.41

### Recrudescent Fever

Recrudescence of fever, the primary outcome, was observed in 65 patients (25.0%) in the overall study population (Table 4). Treatment with low-dose aspirin was not associated with an increase in fever recrudescence vs high-dose aspirin (39 [27.5%] vs 26 [22.0%]; odds ratio [OR], 1.34; 95% CI, 0.76-2.37; *P* = .31). After adjusting for age less than 1 year and incomplete KD, variables that remained in the model after backward selection, the odds of recrudescent fever remained similar between the 2 treatment groups (OR, 1.63; 95% CI, 0.89-2.97; *P* = .11).

**Table 4.** Outcomes of Study Population

Outcome	Overall (N = 260)	Low-Dose (n = 142)	High-Dose (n = 118)	Unadjusted OR (95% CI)^a^	*P* Value	Adjusted OR (95% CI)	*P* Value
Recrudescent fever, No. (%)	65 (25.0)	39 (27.5)	26 (22.0)	1.34 (0.76-2.37)	.31	1.63 (0.89-2.97)	.11^b^
Coronary artery abnormality, No. (%)^c^	20/242 (8.3)	10/135 (7.4)	10/107 (9.4)	0.86 (0.48-1.55)	.62	1.02 (0.55-1.87)	.96^d^
Length of hospital stay, median (IQR), d	3.0 (3.0-5.0)	3.0 (3.0-5.0)	3.0 (3.0-5.0)	NA	.27	NA	.56^e^

Abbreviations: IQR, interquartile range; NA, not applicable; OR, odds ratio.

^a^ High-dose aspirin is the reference.

^b^ Adjusted for age younger than 1 year and incomplete Kawasaki disease using regression modeling.

^c^ Any abnormality at 2- or 6-week follow-up. Eighteen patients did not have follow-up echocardiograms.

^d^ Adjusted for incomplete Kawasaki disease using regression modeling.

^e^ Ajusted for platelet count, fever duration, and incomplete Kawasaki disease.

We performed a stratified analysis for children with complete and incomplete KD. In 167 children with complete KD, treatment with low-dose aspirin, after adjusting for age less than 1 year, was associated with nearly a 2-fold increase in recurrent fever, but this difference did not reach statistical significance (OR, 1.87, 95% CI, 0.82-4.23; *P* = .14). In 93 children with incomplete KD, we found no association of recurrent fever and dosing regimens (OR, 1.33; 95% CI, 0.53-3.36; *P* = .54). In addition, to investigate for an interaction between aspirin dosing and KD type (complete vs incomplete), we added an interaction term to the model and did not find a difference in recrudescent fever between dosing groups.

### Coronary Artery Abnormality and Hospital Length of Stay 

With respect to the secondary outcomes, of the total study population, 8.3% had a coronary artery abnormality at follow up and the median (IQR) length of hospitalization was 3 (3-5) days. Aspirin dose was not associated with an increase in coronary artery abnormalities at follow-up, as the proportion of patients with coronary artery abnormalities in both groups (7.4% in the low-dose group and 9.4% in the high-dose group) was similar (OR, 0.86; 95% CI, 0.48-1.55; *P* = .62). After adjusting for incomplete KD, there was no association between coronary artery abnormalities and treatment group (OR, 1.02; 95% CI, 0.55-1.87; *P* = .96). Stratified by KD type, there was no association between coronary artery abnormalities and treatment group for either complete KD (OR, 1.28; 95% CI, 0.53-3.08; *P* = .58) or incomplete KD (OR, 0.81; 95% CI, 0.34-1.92; *P* = .63). The median (IQR) length of hospital stay for both the low-dose and high-dose groups was 3 (3-5) days (*P* = .27) and remained unchanged after multivariable adjustment (adjusted for platelet count, fever duration, and incomplete KD).

## Discussion

The major findings of this study are that treatment with low-dose aspirin (<10 mg/kg/d) along with high-dose intravenous immunoglobulin within 10 days of symptoms in children with KD was not associated with an increase in recrudescent fever compared with children treated with high-dose (≥10 mg/kg/d) aspirin. In addition, there was no association between aspirin dose and length of hospital stay or coronary artery abnormalities at follow-up in children with KD. When stratified by KD type, initial treatment with low-dose aspirin in children with complete KD was associated with a nearly 2-fold higher odds of recrudescent fever, although this increase was not statistically significant. To our knowledge, this study is unique in investigating aspirin dosing and fever recrudescence within a single US institution and is the largest cohort of such patients.

During the acute, inflammatory phase of KD, the goal of treatment is to decrease systemic inflammation and prevent or reduce vascular damage and thrombosis. To that end, for more than 3 decades, potent anti-inflammatory medications—high-dose intravenous immunoglobulin and aspirin—have been a mainstay of initial KD treatment.^15,16^ The role and mechanisms by which these 2 medications work in KD remain elusive. Despite this uncertainty, there is good evidence regarding optimal dosing and efficacy of intravenous immunoglobulin in reducing coronary artery abnormalities.^8,15,16^ The utility and optimal dosing for aspirin, however, is less clear.^3,4,9^ As such, substantial variation exists in the dosing and duration of aspirin around the world.^1^ While there is evidence that aspirin dosing does not affect the rate of coronary artery abnormalities,^9^ the effect on recrudescent fever, especially in a US population, has not been well studied and has potential ramifications on the need for retreatment, length of stay, and cost. In addition, although high-dose aspirin is generally well tolerated in children, reports of adverse effects, including gastrointestinal bleeding and Reye syndrome, have been documented.^17,18^ Low-dose aspirin, meanwhile, has not been associated with Reye syndrome,^1^ is administered once daily, and may be better tolerated. Given these potential advantages, investigation into the outcomes of children treated with low-dose aspirin is important.

The findings in this study support a growing body of literature suggesting that initial treatment with low-dose aspirin may not be associated with poor outcomes in children with KD.^5,9,19^ One explanation is that the major anti-inflammatory effects in the initial KD treatment regimen come from intravenous immunoglobulin, which sufficiently decreases systemic inflammation regardless of aspirin dosing. Another possibility is that low-dose aspirin, although thought to mainly work on platelet function, has enough anti-inflammatory properties to decrease the odds of fever recrudescence similar to high-dose aspirin. Further investigation into the mechanisms by which intravenous immunoglobulin and aspirin work to decrease systemic inflammation in KD is needed.

Others have looked at the association between aspirin dosing and recrudescence fever in children with KD^3,4,5,6,14^; however, the lack of control groups, differing intravenous immunoglobulin regimens, and multiple treatment centers have limited the conclusions that can be drawn. As a large, tertiary care children’s hospital in the United States serving a diverse patient population with a large number of cases of KD per year, our group is well positioned to conduct comparative research in this population. Before 2016, there was substantial variation in aspirin dosing within our center. After 2016, our group came to consensus on treating patients with KD initially with high-dose (80-100 mg/kg/d) followed by medium-dose (30-50 mg/kg/d) aspirin. Thus, a strength of our study is the large number of patients available for evaluation within a single US center, eliminating the potential bias by treatment center and potentially increasing generalizability among US centers.

### Limitations

This study has limitations. After a large number of patients were excluded, our study was powered to detect an absolute risk difference of 11% or more (OR, ≥1.75). Thus, smaller differences in fever recrudescence would not be detected between treatment groups if present. Also, noting that treatment with low-dose aspirin in children with complete KD was associated with nearly a 2-fold increase in fever recrudescence, it may be that patients who truly have KD, rather than those with an unidentified, alternative diagnosis but treated for KD, have a decreased risk of fever recrudescence when treated with high-dose aspirin. Given the challenges in diagnosis, especially for incomplete KD, as well as the imbalance in KD type between treatment groups in our study, this association was not seen. A larger sample size would be required to determine whether a more subtle, but potentially clinically relevant, difference in fever recrudescence between treatment groups is present.

In addition, as with any retrospective cohort study, it can be difficult to control for differences between treatment groups. Although the only significant clinical difference between treatment groups was the number of incomplete KD cases, which was controlled for during regression analysis, other variables could have the potential to confound the results. Because most patients treated with low-dose aspirin were diagnosed prior to 2016, secular trends in treatment have the potential to introduce bias. With that said, other than aspirin dosing, KD treatment in our group remained consistent during the study period, with many of the same clinicians providing care throughout the study. Also, patient selection via *ICD-9* and *ICD-10* codes, although common for this type of study, has the potential to exclude patients with KD that is not coded properly. Given the large number of patients we excluded owing to inappropriate coding, this issue is a concern, but should not skew to one treatment group over the other.

## Conclusions

Initial treatment with low-dose aspirin, along with high-dose intravenous immunoglobulin, in children with KD did not appear to be associated with an increase in fever recrudescence or coronary artery abnormalities. Given the potential advantages, low-dose aspirin is an attractive option as initial therapy for KD; however, future studies powered to detect more subtle, yet possibly clinically relevant, differences in fever recrudescence are warranted.

## Notes

Supplement. **eFigure.** Residual Plots from Log(LOS) = Dose + Platelets + Fever Duration + Complete KD
