# Supplementary material for: Comparison of Risk of Recrudescent Fever in Children With Kawasaki Disease Treated With Intravenous Immunoglobulin and Low-Dose vs High-Dose Aspirin
Source: JAMA Netw Open. 2020 Jan 3;3(1):e1918565. doi: 10.1001/jamanetworkopen.2019.18565 (PMC6991313; doi:10.1001/jamanetworkopen.2019.18565)
Supplement: Supplement. — eFigure. Residual Plots from Log(LOS) = Dose + Platelets + Fever Duration + Complete KD [file jamanetwopen-3-e1918565-s001.pdf]

Supplementary Online Content

Platt B, Belarski E, Manaloor J, et al. Comparison of risk of recrudescent fever in children with Kawasaki disease treated with intravenous immunoglobulin and low-dose vs high-dose aspirin. *JAMA Netw Open*. 2020;3(1):e1918565. doi:10.1001/jamanetworkopen.2019.18565

eFigure. Residual Plots from Log(LOS)= Dose + Platelets + Fever Duration + Complete KD

This supplementary material has been provided by the authors to give readers additional information about their work.

© 2020 Platt B et al. *JAMA Network Open.*

eFigure. Residual Plots from Log(LOS)= Dose + Platelets + Fever Duration + Complete KD

a.

Residual Plot for Assessing Variance of Homogeneity Residual Plots from Log(LOS)= Dose + Platelets + Fever Duration + Complete KD

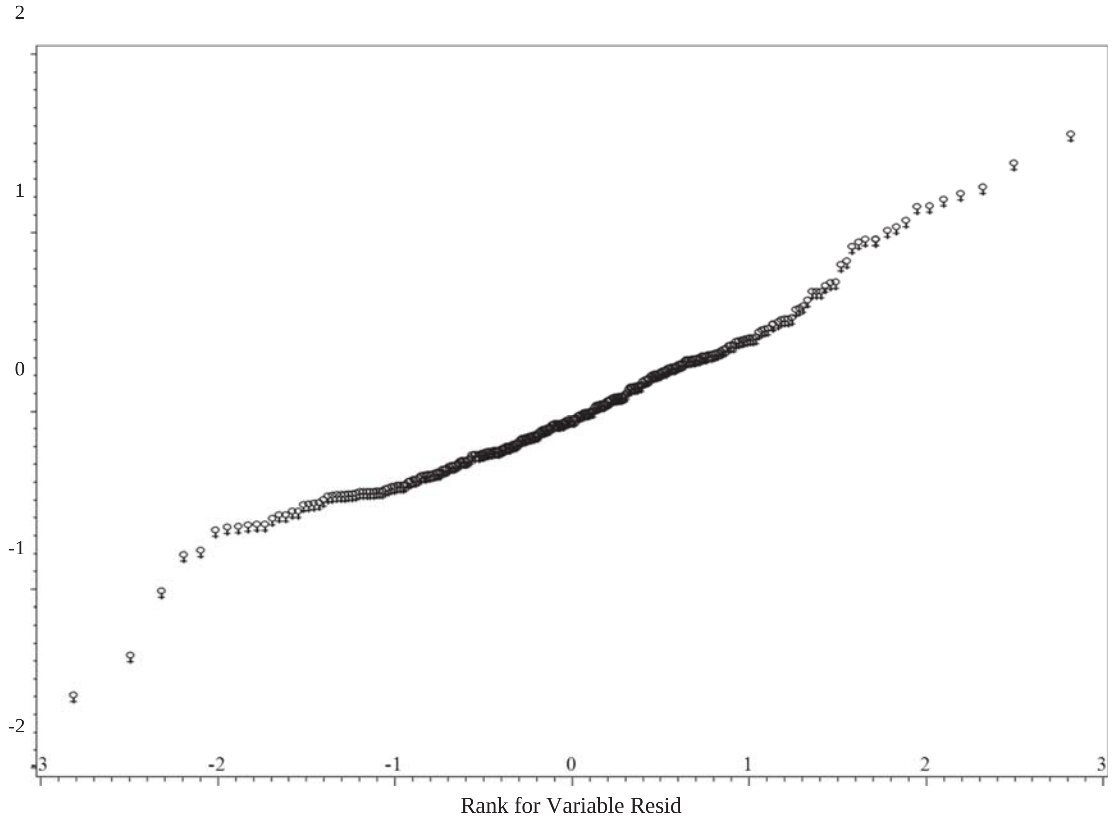

© 2020 Platt B et al. *JAMA Network Open.*

b. **Residual Plot for Normality** Residual Plots from Log(LOS)= Dose + 2 Platelets + Fever Duration + Complete KD

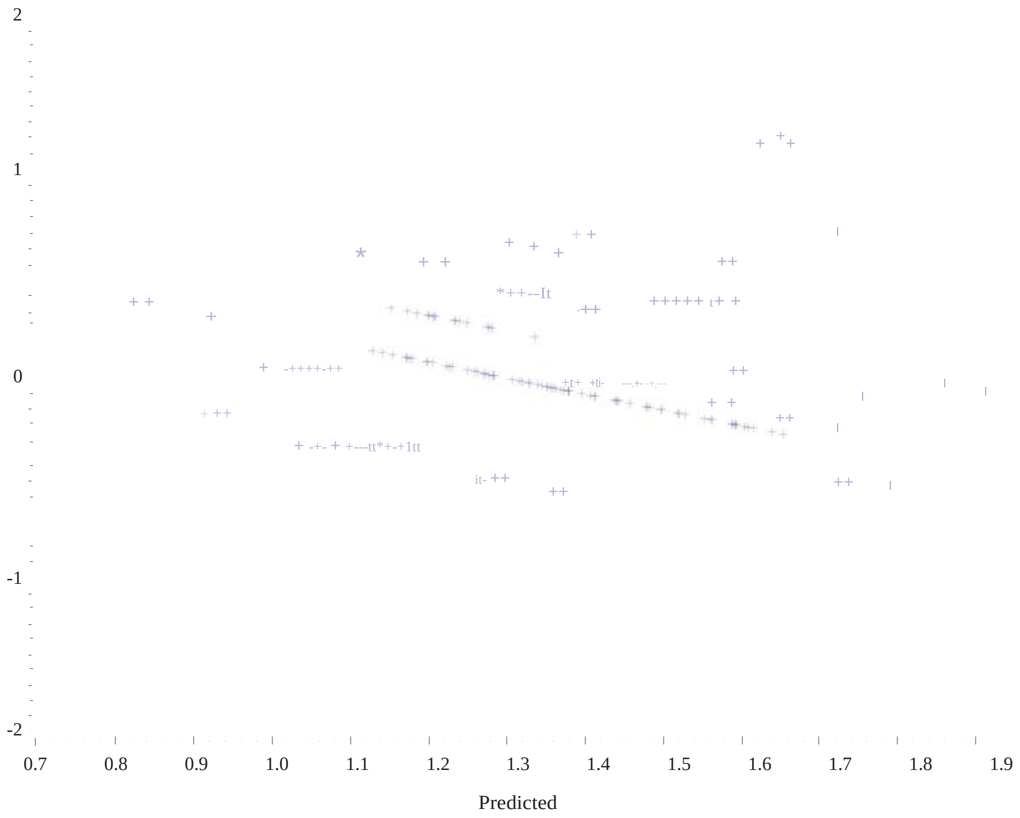

© 2020 Platt B et al. *JAMA Network Open.*
